# Mediterranean Diet Combined With a Low-Carbohydrate Dietary Pattern in the Treatment of Overweight Polycystic Ovary Syndrome Patients

**DOI:** 10.3389/fnut.2022.876620

**Published:** 2022-04-04

**Authors:** Shanshan Mei, Jie Ding, Kaili Wang, Zhexin Ni, Jin Yu

**Affiliations:** ^1^Shanghai University of Traditional Chinese Medicine, Shanghai, China; ^2^Department of Gynecology of Traditional Chinese Medicine, Changhai Hospital of PLA Military Medical University, Shanghai, China; ^3^International Peace Maternity and Child Health Hospital, School of Medicine, Shanghai Jiao Tong University, Shanghai, China

**Keywords:** mediterranean diet, low-carbohydrate diet, polycystic ovary syndrome, overweight mediterranean diet, overweight

## Abstract

**Objectives:**

To determine the therapeutic effect of a Mediterranean diet (MED) combined with a low-carbohydrate (LC) dietary model in overweight polycystic ovary syndrome (PCOS) patients.

**Methods:**

In this 12-week randomized controlled clinical trial, 72 overweight patients with PCOS were randomly assigned to one of two energy-restricted dietary models: the MED/LC diet or the Low fat (LF) diet. After the intervention, the number of the two groups returned to normal menstruation was counted. Body weight, body mass index (BMI), waist circumference, waist-hip ratio (WHR), body fat percentage (BF%), serum fasting insulin(FINS), fasting plasma glucose(FPG), insulin resistance index (HOMA-IR), quantitative insulin sensitivity index (QUIKI), total cholesterol (TC) and high density lipoprotein cholesterol (HDL-C), low density lipoprotein cholesterol (LDL-C), triglyceride (TG), total testosterone (TT), luteinizing hormone (LH), follicle-stimulating hormone (FSH), and prolactin (PRL) were compared between 2 groups before and after intervention.

**Results:**

MED/LC group had more significant reduction trend in weight (−6.10 ± 1.52 kg vs −4.79 ± 0.97 kg, *P* < 0.05), BMI (−2.12 ± 0.57 kg/m^2^ vs −1.78 ± 0.36 kg/m^2^, *P* < 0.05), WC (−6.12 ± 5.95 cm vs −3.90 ± 1.58 cm, *P* < 0.05), WHR (−0.06 ± 0.02 vs −0.03 ± 0.02, *P* < 0.05), BF% (−2.97% ± 1.78% vs −1.19% ± 0.91%, *P* < 0.05), TT (−0.20 ± 0.24 ng/mL vs 0.08 ± 0.11 ng/Ml, *P* < 0.001), LH (−5.28 ± 3.31 mIU/mL vs −3.39 ± 3.64 mIU/mL, *P* < 0.05), and LH/FSH (−1.18 ± 0.75 vs -0.66 ± 1.05, *P* < 0.05) compared with the LF group. In addition, FPG (0.05 ± 0.38 mmol/mL vs -0.50 ± 1.01 mmol/mL, *P* < 0.001), FINS (−4.88 ± 6.11 μU/mL vs −8.53 ± 5.61 μU/mL, *P* < 0.01), HOMA-IR index (−1.11 ± 1.51 vs −2.23 ± 0.25, *P* < 0.05), and QUIKI index (0.014 ± 0.016 vs 0.028 ± 0.019, *P* < 0.05) decreased significantly in the MED/LC group compared with the LF group. Comparing the changes in lipid parameters between the two groups (LF vs MED/LC), significant differences in TG (−0.33 ± 0.32 mmol vs −0.76 ± 0.97 mmol, *P* < 0.05), TC (−0.40 ± 1.00 mmol vs −1.45 ± 2.00 mmol, *P* < 0.05), and LDL-C (−0.41 ± 1.05 mmol vs −0.73 ± 0.76 mmol, *P* < 0.05) were observed.

**Conclusion:**

The results of this study suggest that the MED/LC diet model is a good treatment for overweight PCOS patients, significantly restoring their menstrual cycle, improving their anthropometric parameters and correcting their disturbed endocrine levels, and its overall effectiveness is significantly better than the LF diet model. Therefore, this study recommends that the MED/LC diet model can be used in the clinical treatment of patients with overweight PCOS.

## Introduction

Polycystic ovary syndrome (PCOS) is a common group of endocrine metabolic disorders in women of reproductive age, characterised by polycystic ovarian changes, sporadic ovulation and menstrual disorders (1). Based on conservative estimates, the prevalence of PCOS in women of reproductive age is 6–15%, whereas insulin resistance (IR) is present in 50–70% of patients with PCOS, with obese PCOS patients accounting for approximately 75% of IR patients (2). PCOS causes reproductive endocrine disorders in the near term, which can lead to metabolic syndromes in the long term, such as fatty liver, type 2 diabetes, cardiovascular risk, and increased risk of endometrial cancer (3). Amongst these disorders, IR is an important mechanism causing abnormal glucolipid metabolism and reproductive dysfunction (4), whereas obesity increases IR and compensatory hyperinsulinemia (HI), which in turn increases adipogenesis and decreases lipolysis, promotes ovarian androgen production and exacerbates ovarian androgen hypersecretion disorder, thereby further amplifying reproductive endocrine disorders and metabolic problems in patients with PCOS (5).

Pharmacological treatment; lifestyle management such as diet and exercise and psychological modification have been recommended as the first line of treatment for PCOS at all times (6), with dietary modification playing an important role in the treatment of patients with PCOS patients. Based on previous reports, the diet of patients with PCOS tends to be high in carbohydrates and fat, which to a certain extent increases the IR and lipid inflammatory environment of patients (7) and promotes the progression of the disease. Therefore, calorie-restricted dietary regimens have been recognised as important dietary options for patients with PCOS (8), and corresponding studies on the effects of dietary modification on PCOS are found, such as low-fat (LF) diet, low-carbohydrate (LC) diet, top hypertension diet, pulse-based diet, high-protein diet, ketogenic diet and other dietary models (9–11). At present, considerable research has been conducted to demonstrate that LF diets can effectively reduce central obesity in patients by affecting their insulin levels and lipid metabolism (12, 13). LC diet has also been shown to improve insulin sensitivity and promote normal lipid metabolism (14), however, effective comparisons of dietary regimens and strategies for optimal dietary combinations are not yet well researched.

The Mediterranean diet (MED) model has been studied and discussed in many fields in recent years as a respected dietary structure (15, 16). Its nutritional structure is modelled by emphasising a high intake of vegetables, fruits, fish, seafood, legumes, and nuts, with whole grains generally recommended as staple foods, and cooking with vegetable oils (containing unsaturated fatty acids) instead of animal oils (containing saturated fatty acids), with olive oil being particularly advocated. This diet is more liberal with regard to fat intake, and cereals have a moderating effect on IR as good-quality carbohydrates (17). Based on previous studies, the MED model can reduce the secretion of pro-inflammatory adipocytokines by reducing visceral fat, with a diet structure based on plant-based food intake, and phenolic compounds of plant origin can modulate insulin action and metabolism in insulin-sensitive tissues, with potential preventive or therapeutic effects on IR and IR-related diseases. The increased intake of seafood suggested in the protocol can remarkably increase the levels of n-3 long-chain polyunsaturated fatty acids in human and the circulating levels of insulin-sensitive hormone lipocalin to a certain extent, and the regular intake of seafood can reduce energy intake by 4–9% compared with the regular intake of terrestrial meat, which could be effective in preventing obesity and maintaining energy balance (18, 19). By contrast, the extent to which the MED model is effective has not been supported by additional data in studies of dietary models of PCOS. Thus, this study aimed to combine the MED model with a LC dietary model based on energy restriction, compared with a LF dietary model, investigate whether this novel dietary model could provide significant improvements in reproductive endocrine and metabolic levels in overweight patients with PCOS through a 12-week strict dietary intervention and provide better supplements and recommendations for the behavioural treatment of PCOS in clinical practice.

## Materials and Methods

### Study Design

This study is an open-label, parallel-group randomised controlled trial design for a 12-week intervention with a 4-week follow-up. The study protocol was approved by the by the Ethics Committee of Changhai Hospital of PLA Military Medical University. The study protocol was in accordance with all the principles of the World Medical Association Declaration of Helsinki. Written informed consent was signed by participants prior to their inclusion in the study. The trial was registered at https://ClinicalTrials.gov/, No. CHiECRCT-20160050. Participants had basic information collected through a questionnaire at the beginning of the study, including age, height, weight, medical history, blood pressure, and heart rate. They were randomly assigned to one of two calorie-restricted diets throughout the study period: a LF diet or a Mediterranean/low-carbohydrate (MED/LC) diet. Items collected before and after the intervention included fasting weight on the day of collection, body mass index (BMI), waist circumference (WC), hip circumference (HC), body fat percentage (BF%), fasting plasma glucose (FPG), fasting insulin (FINS), total testosterone (TT), luteinising hormone (LH), follicle-stimulating hormone (FSH), oestradiol (E2), prolactin (PRL), progesterone (P), triglycerides (TG), total cholesterol (TC), high-density lipoprotein cholesterol (HDL-C), and low-density lipoprotein cholesterol (LDL-C).

### Participants

The 72 participants in this study were successively drawn from patients with PCOS, who were attending the Department of Traditional Chinese Medicine and Gynecology at the Changhai Hospital of PLA Military Medical University from 2018 to 2020, with all data collection completed in February 2021. The inclusion criteria were as follows: (a) diagnosis of PCOS according to the Rotterdam criteria (20), (b) age 16–45 years, and (c) BMI consistent with an overweight diagnosis of BMI ≥24.0 kg/m^2^. The exclusion criteria were as follows: (a) combination of other endocrine etiological disorders (congenital adrenal hyperplasia, Cushing’s syndrome, androgen-secreting tumours, hyperprolactinemia, diabetes mellitus, thyroid, adrenal, and other endocrine disorders) according to the Rotterdam criteria; (b) combination of cardiovascular and cerebrovascular diseases, haematological disorders, hepatic and renal insufficiency, and other serious diseases; (c) pregnant women, breastfeeding women, and pregnancy preparation or no contraception during the intervention period; (d) suffering from mental illness or cancer; and (e) have taken non-progesterone hormonal medication or insulin sensitisers or medication affecting lipid metabolism (fish oil, etc.) within 3 months. The exclusion and detachment criteria were as follows: (a) poor compliance during the intervention and non-cooperation with treatment and (b) incomplete observation case information. Participant groupings were randomly assigned by an associate in the department based on a computer-generated random number table.

### Diet Intervention

Both diets aimed at moderate long-term metabolic improvement and restricted the intake of sweets and foods containing trans fatty acids. The participants were divided into two groups, and they joined separate diet management groups, where a professional dietitian recommended 7-day recipes. The LF group had less than 30% of total dietary calories from fat, less than 40 g of fat intake throughout the day and up to 10% saturated fat (21). They do not consume fatty foods such as fatty meats, butter, offal, fried foods, preserved foods, poultry skin, fish roe, shrimp roe, and crabmeat, and they increased the intake of cereals, vegetables, and fruits as appropriate (Supplementary Table 1). In the MED/LC group, which is a combination of MED and LC diet, according to the definition of a LC diet (22), the participants had a maximum carbohydrate intake of less than 20%, a maximum carbohydrate intake of 100 g throughout the day and an increased intake of protein and fat. In combination with the MED group, participants were advised to consume whole grain as a staple food, a high intake of extra virgin olive oil, vegetables (including green leafy vegetables, fruit, cereals, nuts, and legumes), moderate intake of fish and other meat, dairy products, and a low intake of eggs (23) (Supplementary Table 2). The average daily total calorie baseline was maintained for both dietary groups.

### Treatment Adherence

Participants were provided with a daily food recommendation every 7 days via the Chinese social networking software WeChat and were instructed to record their daily food intake from the beginning to the end of the dietary intervention period using the Chinese dietary app Boohee software to calculate the corresponding calories. Participants were asked to perform only daily basal activity during the intervention period and were prohibited from taking any other nutritional supplements. Participants were contacted via WeChat at the end of each week to review the programme, monitor for adverse events and provide counselling support to facilitate the effectiveness of the intervention. All outcome assessments were performed at week 12, and data were collected when normal menstrual cycles returned.

### Anthropometric Measurements and Body Composition Analysis

The height of each participant was measured using an ultrasonic height-measuring device with an accuracy of 1 cm (BXT-15A, BAXIT, Germany), and the weight and BF% were measured using a body composition analyser with an accuracy of 0.1 kg (T6100A, TEZEWA, United States). In addition, the BMI was automatically read by an app connected to the instrument (kg/m^2^). WC and HC were measured by a fixed researcher using a soft ruler with an accuracy of 1 mm. WC was measured as the midpoint of the line between the lowest point of the rib and the upper edge of the iliac crest, and HC was measured as the horizontal circumference of the most prominent part of the hip towards the back. The waist-to-hip ratio (WHR) was calculated as WC (cm)/HC (cm).

### Blood Exams and Biochemical Assessment

Fasting blood samples were obtained in the morning at 2–5 days of the menstrual cycle (or progesterone withdrawal bleeding) from the median cubital vein, and the hormonal and glucose parameters were detected. Blood samples were collected in BD vacutainers tubes (SSTTM II Advance, REF 367958) and centrifuged at 3,500 rpm for 15 min at 4°C, and then serum and plasma were frozen at −80°C. FINS, FSH, LH, E2, TT, PRL, and P were measured by enzyme-linked immunosorbent assay (Elisa Kit, CUSABIO, Wuhan, China). FPG, TC, TG, HDL-C, and LDL-C were measured by coupled enzyme assay (Sigma, United States). The HOMA-IR index was calculated as *FINS*(μU/*mL*)×*FPG*(*mmol*/L)/22.5, and the QUIKI index was calculated as 1/(*log*(*FINS*(μU/*mL*))+*log*(*FPG*(*mg*/*dL*))).

### Statistical Analysis

The Shapiro–Wilk test was used to test whether the data conformed to a normal distribution. Data that conformed to a normal distribution were expressed as mean ± standard deviation (mean ± SD), and data before and after the intervention were counted using paired *t*-tests and between two groups using independent sample *t*-tests. Data that did not conform to a normal distribution were presented as median (25th–75th percentile) and analysed using the Mann–Whitney U test to compare the parameters before and after 12 weeks. All data analyses were performed using SPSS, version 26 (SPSS Inc., Chicago, IL, United States). All data graphs were produced and analysed using GraphPad Prism (version 9.0.0). Differences were considered statistically significant when *P* < 0.05. For the treatment of missing values, these values were not included in this analysis.

## Results

### Participants’ Characteristics

**FIGURE 1:**
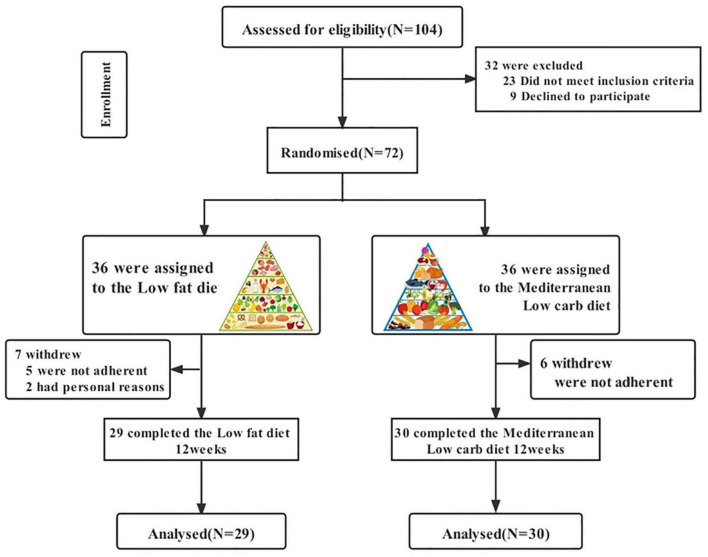
Flow chart from the study design.

Figure 1 shows a flow chart of participants’ progress through the various stages of the study. We recruited a total of 104 patients, of whom 16 were taking hormone medication five had abnormal liver function, two were on insulin sensitisers, six were preparing for pregnancy and three were resistant to diet modification. We included 72 patients in the study programme, of which some patients in both groups did not complete the study cycle because they were unable to adhere to the diet programme or for personal reasons (change of pregnancy plans or outright discontinuation of the study programme), resulting in 29 final completers in the LF diet group and 30 final completers in the MED/LC diet group. No side effects were observed during the dietary intervention in patients with PCOS throughout the study. Based on the initial randomisation, no differences were observed between the LF and MED/LC groups at baseline with regard to age, anthropometrics, sex hormone levels, and blood biochemistry (Table 1).

**TABLE 1:** Baseline characteristics of the study participants.

Characteristic	LF group (N = 29)	MED/LC group (N = 30)	*P*-value
Age (years)	28.07 ± 7.126	27.97 ± 5.295	0.95
Height (m)	1.64 ± 0.05	1.64 ± 0.05	0.87
Weight (kg)	79.76 ± 9.71	79.34 ± 7..94	0.85
BMI (kg/m^2^)	29.57 ± 2.48	29.37 ± 2.22	0.75
Waist circumference (cm)	96.66 ± 9.98	96.05 ± 10.27	0.82
Hip circumference (cm)	105.3(101.95–114.75)	105.15(101.48–110.13)	0.59*
WHR	0.90 ± 0.05	0.91 ± 0.05	0.25
body fat percentage (BF%)	37.28 ± 5.19	37.41 ± 7.03	0.94
TT (ng/ml)	0.85(0.80–0.94)	0.89(0.80–1.02)	0.28*
LH (mIU/ml)	8.11(6.87–11.09)	9.07(7.12–11.28)	0.51*
FSH (mIU/ml)	4.58 ± 1.36	4.70 ± 1.28	0.75
LH/FSH ratio	1.82(1.55–2.52)	2.02(1.64–2.27)	0.58*
PRL (ng/ml)	11.28(7.91–15.39)	12.36(9.36–16.57)	0.41*
FPG (mmol/ml)	5.12(4.77–5.44)	5.32(4.95–5.62)	0.22*
FINS (μU/ml)	18.90(14.80–23.45)	19.80(14.93–27.40)	0.53*
HOMA-IR index	4.00(3.39–5.43)	4.90(3.70–6.75)	0.19*
QUIKI index	0.307 ± 0.014	0.304 ± 0.013	0.30
TG (mmol)	1.48(1.17–2.60)	1.67(1.02–2.14)	0.92*
TC (mmol)	4.99(4.20–5.40)	5.05(4.50–5.76)	0.50*
HDL-C (mmol)	1.21(0.96–1.37)	1.09(0.95–1.25)	0.28*
LDL-C (mmol)	2.86(2.21–3.60)	3.06(2.66–3.52)	0.30*

BMI, body mass index; WHR, waist-to-Hip Ratio; BF%, body fat percentage TT, total testosterone; LH, luteinizing hormone; FSH, follicle stimulating hormone; LH/FSH, ratio of luteinizing hormone to follicle stimulating hormone; PRL, prolactin; FPG, fasting plasma glucose; FINS, fasting insulin; HOMA-IR, homeostatic model assessment of insulin resistance; QUIKI, quantitative insulin sensitivity check index; TC, total cholesterol; TG, triglyceride; HDL-C, high-density lipoprotein cholesterol; LDL-C, low-density lipoprotein cholesterol. P Comparison between two groups has been assessed using independent t-test. Results are expressed as mean ± SD. *P Comparison between two groups has been assessed using Mann–Whitney tests.

*Results are expressed as median (25th–75th percentile).*

### Dietary Intake

**FIGURE 2:**
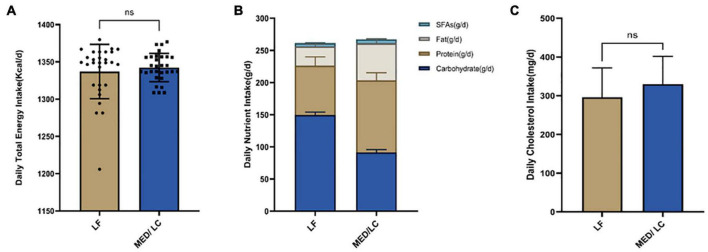
Intake of energy and nutrients between the diet intervention groups. **(A)** Baseline for daily total energy intake remained the same for both groups(*P* > 0.05). **(B)** The Mediterranean/low-carbohydrate (MED/LC) diet more greatly decreased the intake of carbohydrate and more greatly increased the intake of protein, fat and saturated fatty acids (SFAs). **(C)** No difference in daily cholesterol intake between the two groups(*P* > 0.05). Analysis of variance with a covariance (ANCOVA) test was used. LF, low fat diet.

Differences in total nutrient intake were calculated on three randomly selected days based on participants’ dietary records throughout the intervention (Figure 2). No statistically significant differences were found between the two groups with regard to total energy and cholesterol intake. The MED/LC group had a relatively lower carbohydrate intake (*P* < 0.001), a relatively higher total protein and fat intake (*P* < 0.001), and a significant difference in saturated fatty acid intake (*P* < 0.001) compared with the LF group (Table 2).

**TABLE 2:** Dietary intakes of study participants throughout the study.

	LF Group (N = 29)	MED/LC Group (N = 30)	*P*-value
Total energy (Kcal/day)	1348.79(1318.94–1363.40)	1342.34(1333.90–1356.91)	0.69*
Carbohydrate (g/day)	149.61(145.39–153.60)	91.73(88.44–95.23)	<0.001*
Protein (g/day)	76.64 ± 13.62	112.35 ± 11.72	<0.001
Fat (g/day)	29.79 ± 3.02	57.51 ± 6.56	<0.001
SFAs (g/day)	5.26 ± 0.54	6.08 ± 0.94	<0.001
Cholesterol (mg/day)	296.27 ± 75.64	329.97 ± 71.59	0.08

*SFAs, saturated fatty acids; LF, low fat diet; MED/LC, Mediterranean/low-carbohydrate diet. P Comparison of between-group changes (independent t-test).*

Data are expressed as mean ± SD. *P Comparison of between-group changes (Mann–Whitney tests). Results are expressed as median (25th–75th percentile).

### Menstruation Situation

At the end of the study, the number of patients who returned to normal menstruation in the two groups was counted. 72.4% (21/29) of patients in the LF group returned to normal menstrual cycles, whereas 86.7% (26/30) of patients in the MED/LC group returned to normal menstrual cycles, with no significant difference between the two groups (*P* = 0.174 > 0.05).

### Anthropometric and Body Composition Measurements

**FIGURE 3:**
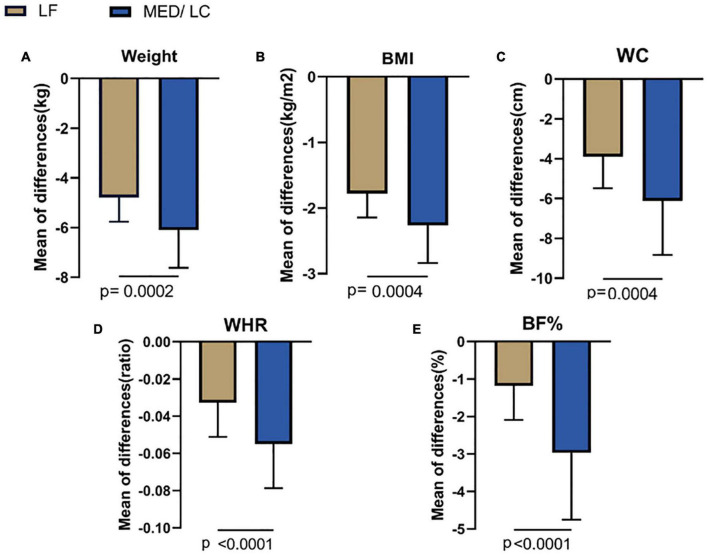
Comparison of changes in anthropometric variables between two groups. **(A)** The MED/LC diet resulted in more weight loss than the LF diet. **(B)** MED/LC diet reduced body mass index (BMI) more than LF diet. **(C)** The MED/LC diet resulted in more waist circumference (WC) loss than the LF diet. **(D)** MED/LC diet reduced waist-to-hip ratio (WHR) more than LF diet. **(E)** The MED/LC diet resulted in more body fat percentage (BF%) loss than the LF diet. Data are mean (± SD). Analysis of variance with a covariance (ANCOVA) test was used.

Combining the data before and after the dietary intervention in both groups, a decrease in weight, BMI, WC, WHR, and BF% in both groups was observed, with statistically significant differences (*P* < 0.05 or *P* < 0.001; Table 3), indicating that both interventions had effective management effects. Figure 3 shows the data before and after the two groups showed that the MED/LC group had more significant reduction trend in weight (−6.10 ± 1.52 kg vs −4.79 ± 0.97 kg), BMI (−2.12 ± 0.57 kg/m^2^ vs −1.78 ± 0.36 kg/m^2^), WC (−6.12 ± 5.95 cm vs −3.90 ± 1.58 cm), WHR (−0.06 ± 0.02 vs −0.03 ± 0.02), and BF% (−2.97% ± 1.78% vs. −1.19% ± 0.91%) compared with the LF group.

**TABLE 3:** Anthropometric measurements and body composition at baseline and after the intervention.

	LF group (n = 29)			MED/LC group (n = 30)		
		
	LF before	LF after	*P*-value	MED/LC before	MED/LC after	*P*-value
Weight (kg)	76.8(71.85–86.55)	72.5(67.5–81.35)	0.046*	79.34 ± 7.94	73.24 ± 7.12	<0.001
BMI (kg/m^2^)	29.57 ± 2.48	27.78 ± 2.39	<0.001	29.37 ± 0.41	27.11 ± 1.86	<0.001
WC (cm)	96.66 ± 9.98	92.75 ± 9.20	<0.001	96.05 ± 10.27	89.93 ± 9.65	<0.001
WHR	0.90 ± 0.05	0.87 ± 0.05	<0.001	0.91 ± 0.05	0.86 ± 0.05	<0.001
BF (%)	37.28 ± 5.19	36.10 ± 5.30	<0.001	37.41 ± 7.03	34.44 ± 7.67	<0.001

*BMI, body mass index; WC, waist circumference; WHR, waist-to-hip Ratio; BF%, body fat percentage; P Comparison of within-group changes (Paired sample t-test).*

Dates are expressed as mean ± SD. *P Comparison of within-group changes (Mann–Whitney tests). Results are expressed as median (25th–75th percentile).

### Gonadal Parameters

With regard to sex hormones (Table 4), significant changes in TT, LH, and LH/FSH were observed before and after the dietary intervention in both groups (*P* < 0.05 or *P* < 0.001), whereas FSH and PRL were basically unchanged compared with baseline values (*P* > 0.05).

**TABLE 4:** Serum sex hormone levels at baseline and after the intervention.

	LF group (n = 29)			MED/LC group (n = 30)		
		
	LF before	LF after	*P*-value	MED/LC before	MED/LC after	*P*-value
TT (ng/ml)	0.85(0.80–0.94)	0.80(0.71–0.89)	0.03*	0.89(0.80–1.02)	0.72(0.51–0.84)	<0.001*
LH (mIU/ml)	8.11(6.87–11.09)	5.26(2.57–6.89)	<0.001*	9.07(7.12–11.28)	3.59(1.85–5.48)	<0.001*
FSH (mIU/ml)	4.58 ± 1.36	4.40 ± 1.63	0.54	4.72(3.92–5.76)	4.39(3.79–4.96)	0.50*
LH/FSH ratio	1.82(1.55–2.52)	1.24(0.71–1.77)	0.001*	2.03 ± 0.56	0.85 ± 0.09	<0.001
PRL (ng/ml)	11.28(7.91,15.39)	13.52(8.93–17.75)	0.32*	12.36(9.36–16.57)	12.56(9.48–15.51)	0.9*

TT, total testosterone; LH, luteinizing hormone; FSH, follicle stimulating hormone; LH/FSH, ratio of luteinizing hormone to follicle stimulating hormone; PRL, prolactin; P Comparison of within-group changes (Paired sample t-test). Data are expressed as mean ± SD. *P Comparison of within-group changes (Mann–Whitney tests). Results are expressed as median (25th–75th percentile).

**FIGURE 4:**
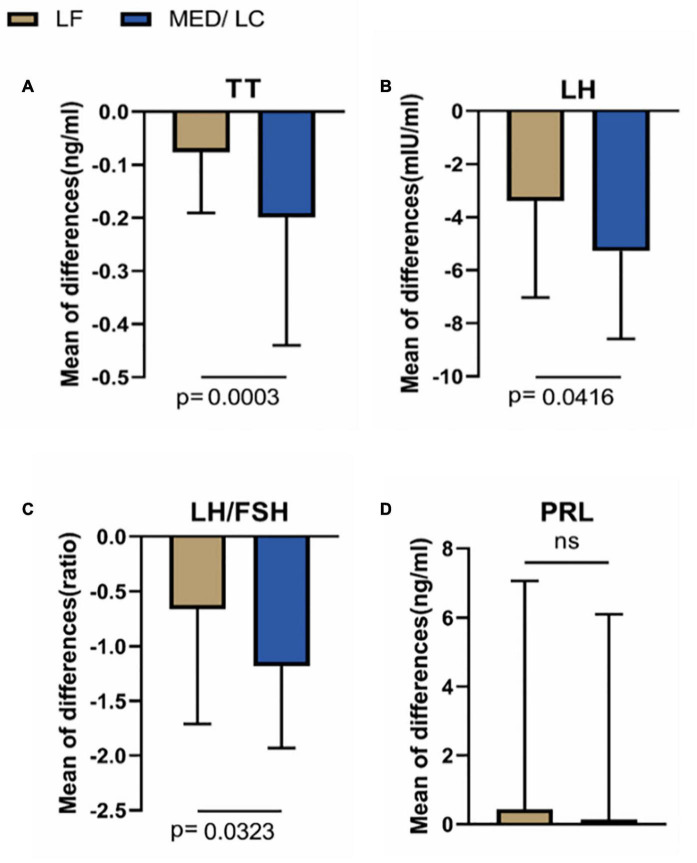
Comparison of changes in serum sex hormone levels between two groups. **(A)** The MED/LC diet resulted in more total testosterone (TT) loss than the LF diet. **(B)** MED/LC diet reduced luteinizing hormone (LH) more than LF diet. **(C)** The MED/LC diet resulted in more ratio of luteinizing hormone to follicle-stimulating hormone (LH/FSH) loss than the LF diet. **(D)** Prolactin (PRL) changes of MED/LC diet and LF diet had no difference. Data are mean(±SD). Analysis of variance with a covariance (ANCOVA) test was used.

As shown in Figure 4, the trend of hormonal changes in the two groups was compared, and a decrease in TT (−0.20 ± 0.24 ng/mL vs. 0.08 ± 0.11 ng/mL) was found in the MED/LC group compared with the LF group (*P* < 0.001). In addition, a decrease in LH (−5.28 ± 3.31 mIU/mL vs. −3.39 ± 3.64 mIU/mL) and LH/FSH (−1.18 ± 0.75 vs −0.66 ± 1.05) was found, and the differences were statistically significant (*P* < 0.05). However, the change in PRL between the two groups was not statistically significant (*P* > 0.05).

### Metabolic Parameters

**FIGURE 5:**
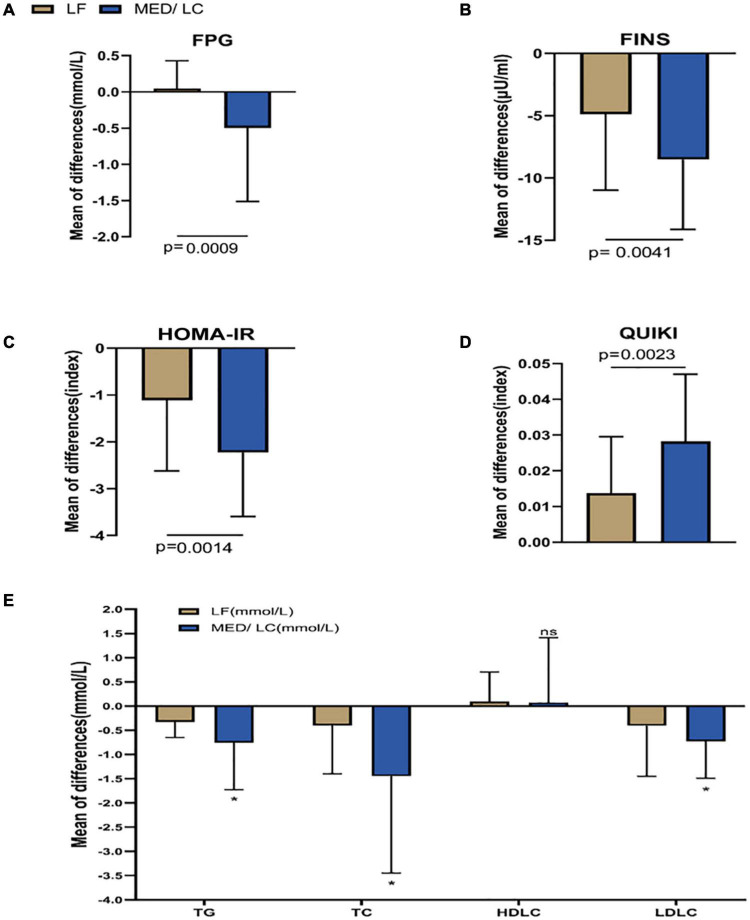
Comparison of changes in metabolic parameters between two groups. **(A)** Change in fasting plasma glucose (FPG) in the MED/LC diet compared to the LF diet. **(B)** Change in fasting insulin (FINS) in the MED/LC diet compared to the LF diet. **(C)** Change in homeostatic mode assessment of insulin resistance (HOMA-IR) in the MED/LC diet compared to the LF diet. **(D)** Change in quantitative insulin sensitivity check index (QUIKI) in the MED/LC diet compared to the LF diet. **(E)** Change in blood lipid level in the MED/LC diet compared to the LF diet. TC, total cholesterol; TG, triglyceride; HDL-C, high-density lipoprotein cholesterol; LDL-C, low-density lipoprotein cholesterol. Data are mean (± SD). Analysis of variance with a covariance (ANCOVA) test was used. *P < 0.05. ns: *P* > 0.05.

After the intervention in both groups (Table 5), FINS, HOMA-IR index, QUIKI index and lipid parameters, including TG, TC, and LDL-C, decreased significantly (*P* < 0.05 or *P* < 0.001), but HDL-C levels did not change significantly (*P* > 0.05). The reduction in blood glucose in the MED/LC group after the intervention was statistically significant (*P* < 0.05). In the MED/LC group, FPG (0.05 ± 0.38 mmol/mL vs -0.50 ± 1.01 mmol/mL, *P* < 0.001), FINS (−4.88 ± 6.11 μU/mL vs −8.53 ± 5.61 μU/mL, *P* < 0.01), HOMA-IR index (−1.11 ± 1.51 vs −2.23 ± 0.25), and QUIKI index (0.014 ± 0.016 vs 0.028 ± 0.019) decreased compared with the LF group (Figure 5), and all differences were statistically significant (*P* < 0.05). Comparing the changes in lipid parameters between the two groups (LF vs MED/LC), significant differences in TG (−0.33 ± 0.32 mmol vs −0.76 ± 0.97 mmol), TC (−0.40 ± 1.00 mmol vs −1.45 ± 2.00 mmol), and LDL-C (−0.41 ± 1.05 mmol vs −0.73 ± 0.76 mmol; *P* < 0.05) were observed, but no significant difference in HDL-C was found between the two groups (*P* > 0.05).

**TABLE 5:** Glucolipid metabolism index at baseline and after the intervention.

	LF group (n = 29)			MED/LC group (n = 30)		
		
	LF before	LF after	*P*-value	MED/LC before	MED/LC after	*P*-value
FPG (mmol/ml)	5.17 ± 0.47	5.22 ± 0.47	0.491	5.32(4.95–5.62)	4.97(4.45–5.38)	0.017*
FINS (μU/ml)	18.90(14.80–23.45)	13.49(9.75–19.45)	0.006*	21.7 ± 7.62	13.18 ± 5.58	<0.001
HOMA-IR	4.00(3.39–5.43)	3.06(2.24–4.33)	0.013*	5.17 ± 1.7	2.94 ± 1.36	<0.001
QUIKI	0.307 ± 0.014	0.321 ± 0.020	<0.001	0.304 ± 0.013	0.332 ± 0.024	<0.001
TG (mmol)	1.48(1.17–2.60)	1.10(0.88–1.94)	0.039*	1.67(1.02–2.14)	1.03(0.76–1.33)	0.003*
TC (mmol)	4.95 ± 1.02	4.55 ± 0.82	0.037	5.05(4.50–5.76)	4.05(2.98–4.82)	<0.001*
HDL-C (mmol)	1.21(0.96–1.37)	1.27(1.05–1.34)	0.355*	1.09(0.95–1.25)	1.14(0.95–1.28)	0.383*
LDL-C (mmol)	2.84 ± 0.88	2.43 ± 0.83	0.046	3.06(2.66–3.52)	2.44(1.91–2.91)	<0.001*

FPG, fasting plasma glucose; FINS, fasting insulin; HOMA-IR, homeostatic model assessment of insulin resistance; QUIKI, quantitative insulin sensitivity check index; TC, total cholesterol; TG, triglyceride; HDL-C, high-density lipoprotein cholesterol; LDL-C, low-density lipoprotein cholesterol; P Comparison of within-group changes (Paired sample t-test). Data are expressed as mean ± SD. *P Comparison of within-group changes (Mann–Whitney tests). Results are expressed as median (25th–75th percentile).

## Discussion

This study was the first to use the MED combined with a LC diet for 12 weeks of strict dietary management in overweight patients with PCOS. It also compared the effects of the MED/LC diet and the LF diet on anthropometric indicators, reproductive endocrine levels, degree of IR and lipid metabolism levels in patients with PCOS; recorded the number of people who returned to normal menstrual cycles during management in both groups and investigated the degree of rationality and effectiveness of the two dietary regimens.

Polycystic ovary syndrome is a common gynaecological endocrine metabolic disorder in adolescent and fertile women. IR is a metabolic abnormality characteristic of PCOS, and it leads to compensatory elevation of body insulin levels and the development of HI, which exacerbates hyperandrogenaemia, anovulation and polycystic ovary formation (24). Most patients with PCOS are formally obese, and obesity is an important cause of IR in patients with PCOS. Clinical management of PCOS is still mostly limited to pharmacological treatment. As research into the mechanisms of IR in patients with PCOS progresses, including its effects on glucose and lipid metabolism and the warning of long-term complications such as cardiovascular disease and endometrial cancer, considerable attention should be given to lifestyle interventions as a safe, effective and closely related daily treatment management modality (25). As the first line of treatment for PCOS, the management of dietary behaviour plays an important role in the efficacy of the disease, and no standardised dietary protocols are available at present. In an earlier clinical study, the average daily total energy intake of patients with PCOS was higher than that of the common people, and limiting their total energy intake ameliorated their weight, androgen secretion and insulin index (26). Subsequently, overweight patients with PCOS have a predominance of visceral adiposity, and they are evidently and centrally obese. In addition, adipose tissue secretes a variety of adipokines, including leptin, lipocalin, resistin and visceral adiponectin to promote the development of PCOS. The formation of obesity is strongly related to the daily preference for high-fat foods (27). The development of obesity is strongly associated with the daily preference for high-fat foods. Therefore, some researchers have developed a LF diet model with no energy restriction and a low glycaemic index diet model to compare the improvement of obesity and reproductive endocrinology in patients with PCOS and found that both dietary models improved obesity in patients with PCOS but not reproductive endocrinology (28). In the pathogenesis of PCOS, IR plays a crucial role as a central mechanism for the sensitivity of body’s glucose metabolism, usually manifested by poor glucose tolerance and reduced insulin sensitivity (29). Therefore, controlling the level of carbohydrate content in the daily dietary intake of patients with PCOS plays an important role. Jafari-Maram et al. found diets lower in carbohydrate and higher in protein and fat were not associated with overweight, obesity and cardiovascular risk factors (30). In a meta-analysis of intervention studies in patients with PCOS using an unrestricted energy LC diet model, LC significantly reduced BMI, androgen and IR levels and improved lipid metabolism in eight relevant RCTs compared with standard dietary regimens (31). Thus, a LC diet is recommended. The MED model, a dietary model that has emerged in recent years, is based on a rich variety of plant foods, including a large number of vegetables, fruits, grains and cereals, legumes, nuts and seeds, with fat accounting for up to 35% of total dietary energy and less than 7–8% of saturated fatty acids. It has a refined broad framework for the composition of the diet, and it is widely used in metabolic and cardiovascular diseases (32), but its application in PCOS is less common. The MED model can effectively modify IR (33). Therefore, in this study, we used the MED structural model combined with the LC diet model, which was compared with the LF diet model, to provide a 12-week dietary intervention for overweight patients with PCOS based on restricted total energy intake, and we found that at the end of the intervention, both dietary models had an effect on the anthropometric indicators of patients with PCOS, reproductive endocrine levels, the degree of IR and lipid metabolism-related indicators. The MED/LC group showed more significant improvements in weight, BMI, WC, WHR, and BF% than the LF diet group. The primary issue for overweight patients with PCOS is weight and body fat loss; therefore, the MED/LC regimen is effective for overweight patients with PCOS, surpassing the LF regimen to a certain extent. With regard to reproductive endocrine levels, TT, LH, and LH/FSH all decreased to a certain extent in the LF group after the intervention, but the decrease was more prominent after the MED/LC intervention. In addition, with regard to FSH and PRL levels, a slight change was observed in both groups. The decrease in these indicators is important for follicular development and the recovery of ovulatory cycles in patients with PCOS. The decline in TT is particularly important. Hyperandrogenaemia, a representative pathological mechanism in PCOS, is a pathological increase in androgens that activates the earliest stages of follicular growth, thereby stimulating the growth of small sinus follicles, leading to the recruitment of small follicles and the formation of polycystic ovaries. Moreover, a high androgen level increases the expression of granulosa cell androgen receptors and FSH receptors, promoting granulosa cell and follicular membrane cell proliferation and cortical thickening, with ovulatory disturbances and secondary menstrual disorders (34). Therefore, a decrease in androgens would play a direct role in restoring ovulation. In previous studies, the LF diet alone did not significantly improve reproductive endocrine disorders in overweight patients with PCOS (28), whereas the LF diet model in this study showed good improvement. The present study controlled the total energy intake by setting the LF diet model, which had an important effect on the metabolic level to a certain extent. This result suggests that the restriction of total energy intake is important in the development of dietary intervention, regardless of the changes in the composition of the diet. Comparing the number of people who returned to normal menstrual cycles between the two groups, the return to normal menstrual cycles was greater than 50% in the LF and MED/LC groups (72.4 vs. 86.7%), with no significant difference between the two groups, indicating that both dietary regimens were effective in restoring ovulation in overweight patients with PCOS. In the dietary regimen of this study, the LF group was set up with an average daily fat intake of less than 40 g, thereby significantly increasing the ratio of carbohydrate to protein intake compared with the MED/LC group with regard to dietary structure. When comparing the reduction in the degree of IR, we found that the FPG in the LF group was higher than that before the intervention, which may be related to the higher proportion of carbohydrate intake in this model. Therefore, the total energy intake and the total fat intake were controlled during the 12 weeks of the protocol, and the FINS, HOMA-IR, and QUIKI indices still showed a decreasing trend to a certain extent, indicating that our LF diet in the presence of calorie restriction can still alleviate the degree of IR, although the carbohydrate intake ratio was correspondingly higher. The results of a previous study found little difference in changes in blood glucose and HI-related indicators after a high-fat diet versus a high-carbohydrate diet, but the metabolic pattern shifted to impaired insulin sensitivity during the high-fat diet (35). This finding suggests that the intake of fat content has a profound effect on the IR status, which could explain the corresponding improvement in IR in our LF diet model. However, the MED/LC group significantly controlled carbohydrate intake compared with the LF group. Combining the two dietary models, the MED/LC group significantly increased protein intake compared with the LF group, whereas fat intake did not significantly exceed the standard diet (the standard for a LF diet is a total daily intake of <50 g, and the fat intake in this study was at 57.51 ± 6.56 g). Therefore, the MED/LC diet model in this study is similar to the high-protein (HP) diet model to a certain extent. In an earlier trial of a dietary intervention for PCOS, researchers found significant improvements in anthropometric indicators and testosterone levels and a slight difference in lipid levels in patients with PCOS compared with the standard ratio dietary model after intervention with a dietary model that simply increased the protein/carbohydrate ratio (36). In studies of metabolic-related diseases, calorie-restricted HP diet model can relieve hyperglycaemia and IR status by modifying intestinal flora (37, 38), and the results of the present study suggest that a similar mechanism may exist in the MED/LC model. Comparing the relevant metabolic indices before and after the intervention of the two dietary models in this study, the decrease in IR-related indices (FPG, FINS, HOMA-IR index, and QUIKI index) and lipid profile (TG, TC, and LDL-C) after the MED/LC diet intervention was significant compared with the LF group, which indicated that the MED/LC diet model has an advantage over the LF diet model with regard to calorie restriction. The MED/LC group had an advantage over the LF group with regard to improvement in all PCOS-related indicators, which was also related to the difference in the specific refinement of food between the two groups. The MED diet recommends a whole-grain diet as the staple food, and the LF model does not emphasise restricting the type of staple food, which may also have some effect. Based on previous studies, the dietary fibre content of cereals is higher than that of refined carbohydrates (e.g., rice and noodles), and the higher the dietary fibre intake, the lower the body weight, glycaemic load and triglyceride and cholesterol levels (39), which would better explain the advantages of the MED/LC dietary model. The MED/LC group is more finely structured than the LF group and is more useful for clinicians to understand its specific application. The strength of this study lies in the combination of the MED diet model and the LC diet model, with specific dietary interventions based on calorie restriction and a comparison with the LF group. Analysis of the results shows that the MED/LC diet model with calorie restriction has a greater advantage in the improvement of all indicators in overweight patients with PCOS, and it provides a better reference for the intervention criteria of the PCOS diet structure. In addition, the MED/LC diet model has a more specific reference for the arrangement of the diet structure, which also facilitates clinical application. Few prospective controlled studies have used the MED diet model for the treatment of PCOS. One single-arm study of the MED diet model combined with a ketogenic diet model showed that the combined diet model was effective in improving PCOS anthropometric indicators, reproductive endocrine levels and IR levels (40). However, the ketogenic diet is not long lasting because of potential negative effects on the kidneys and adverse effects such as decreased insulin sensitivity after withdrawal of ketones (41), which also has some reference value for our study. Notably, this study still has significant limitations similar to other studies of lifestyle interventions. First, patient adherence to the dietary intervention in this study was highly problematic, with PCOS patients having difficulty adhering to a single dietary model for 12 consecutive weeks. Second, this study was a single-centre trial, and the participants were all Chinese patients. The development of the diet plan considered certain regional characteristics, and the generalised type of diet may have some limitations for various food cultures. Third, the treatment period was only 12 weeks, without long-term intervention and follow-up, and some statistical biases against long-term effects were found. Therefore, this study could establish a relatively advantageous dietary model as a model reference for clinicians in the treatment of PCOS with regard to dietary interventions.

## Conclusion

This study evaluated the effectiveness of the MED diet model in combination with the LC diet model in the treatment of overweight patients with PCOS, based on restricting total energy intake, compared with the LF diet model.

The results of the study showed that the LF and MED/LC dietary models were effective in modifying anthropometric parameters, reproductive endocrine levels, IR levels and lipid levels in patients with PCOS, with the MED/LC dietary model being more effective and the recovery of menstrual cycles being approximately the same in both groups. Therefore, the MED/LC diet model was recommended for the treatment of overweight patients with PCOS.

## Data Availability Statement

The raw data supporting the conclusions of this article will be made available by the authors, without undue reservation.

## Ethics Statement

The studies involving human participants were reviewed and approved by the Ethics Committee of Changhai Hospital of PLA Military Medical University. The patients/participants provided their written informed consent to participate in this study.

## Author Contributions

SM and JD collected the data and thesis writing. KW conducted the statistical analyses. ZN revised the final version. JY proposed the research ideas and designed the research plans. All authors contributed to the article and approved the submitted version.

## Conflict of Interest

The authors declare that the research was conducted in the absence of any commercial or financial relationships that could be construed as a potential conflict of interest. The reviewer JQ declared a shared affiliation with the author JY to the handling editor at the time of review.

## Publisher’s Note

All claims expressed in this article are solely those of the authors and do not necessarily represent those of their affiliated organizations, or those of the publisher, the editors and the reviewers. Any product that may be evaluated in this article, or claim that may be made by its manufacturer, is not guaranteed or endorsed by the publisher.

## Abbreviations

PCOS: polycystic ovary syndrome
IR: insulin resistance
HI: hyperinsulinemia
LF: low fat
MED: Mediterranean diet
LC: low carbohydrate
HP: high protein
SFAs: saturated fatty acids
BMI: body mass index
WHR: waist-to-hip ratio
BF%: body fat percentage
TT: total testosterone
LH: luteinising hormone
FSH: follicle-stimulating hormone
LH/FSH: ratio of luteinising hormone to follicle-stimulating hormone
PRL: prolactin
FPG: fasting plasma glucose
FINS: fasting insulin
HOMA-IR: homeostatic model assessment of insulin resistance
QUIKI: quantitative insulin sensitivity check index
TC: total cholesterol
TG: triglyceride
HDL-C: high-density lipoprotein cholesterol
LDL-C: low-density lipoprotein cholesterol.
